# Supplementary material for: Diagnostic accuracy of the aortic dissection detection risk score alone or with D-dimer for acute aortic syndromes: Systematic review and meta-analysis
Source: PLoS One. 2024 Jun 21;19(6):e0304401. doi: 10.1371/journal.pone.0304401 (PMC11192411; doi:10.1371/journal.pone.0304401)
Supplement: S1 Appendix — (DOCX) [file pone.0304401.s001.docx]

**S1 Appendix. The Aortic Dissection Detection Risk Score (ADD-RS)**

The ADD-RS is calculated on the presence of risk markers in the clinical categories of predisposing conditions, pain features and physical findings. The score allocates one point if the patient has a high-risk condition, one point if they have a high-risk symptom, and one point if they have a high-risk examination finding, to give an overall score between zero and three. A threshold of greater than zero or greater than one can then select patients for further investigation.

**Fig S1: The Aortic Dissection Detection Risk Score (ADD-RS)**

| **High-risk conditions** | |
| --- | --- |
| • Marfan syndrome  • Family history of aortic disease  • Known aortic valve disease  • Recent aortic manipulation  • Known thoracic aortic aneurysm | 1 Point if any present |

| **High-risk pain features** | |
| --- | --- |
| Chest, back, or abdominal pain described as:  • Abrupt in onset  • Severe in intensity  • Ripping or tearing in quality | 1 Point if any present |

| **High-risk exam features** | |
| --- | --- |
| • Pulse deficit or systolic BP differential  • Focal neurologic deficit (with pain)  • Murmur of aortic insufficiency (new, with pain)  • Hypotension or shock state | 1 Point if any present |
