# Supplementary material for: Diagnostic accuracy of the aortic dissection detection risk score alone or with D-dimer for acute aortic syndromes: Systematic review and meta-analysis
Source: PLoS One. 2024 Jun 21;19(6):e0304401. doi: 10.1371/journal.pone.0304401 (PMC11192411; doi:10.1371/journal.pone.0304401)
Supplement: S2 Appendix — (DOCX) [file pone.0304401.s002.docx]

**S2 Appendix. Diagnostic pathway for patients presenting with clinical features suggestive of AAS (Canadian guidelines)**

**Fig S2: Diagnostic pathway for patients presenting with clinical features suggestive of AAS (Canadian guidelines)^28^ (Reproduced with permission of the copyright owner)**
