# Supplementary material for: Diagnostic accuracy of the aortic dissection detection risk score alone or with D-dimer for acute aortic syndromes: Systematic review and meta-analysis
Source: PLoS One. 2024 Jun 21;19(6):e0304401. doi: 10.1371/journal.pone.0304401 (PMC11192411; doi:10.1371/journal.pone.0304401)
Supplement: S3 Appendix — (DOCX) [file pone.0304401.s003.docx]

**S3 Appendix. Literature search strategies**

| **Database searched:** | **Ovid MEDLINE(R) Epub Ahead of Print, In-Process & Other Non-Indexed Citations, Ovid MEDLINE(R) Daily, Ovid MEDLINE and Versions(R)** |
| --- | --- |
| **Platform or provider used:** | **Ovid SP** |
| **Date of coverage:** | **1946 to February 2024** |
| **Search undertaken:** | **February 2024** |

1 acute aortic syndrome/

2 aneurysm, dissecting/ or exp aortic aneurysm/

3 (AAS or (aortic syndrome* and acute)).mp.

4 ((aort* adj3 dissect*) and acute*).mp.

5 ((intramural adj3 h?ematoma*) or (intra-mural adj3 h?ematoma*)).mp.

6 ((aort* adj3 ulcer*) and (atherosclero* or penetrat* or symptom*)).mp.

7 1 or 2 or 3 or 4 or 5 or 6

8 (scor* or biomarker* or D-dimer*).mp.

9 clinical decision rules/

10 clinical decision rule*.mp.

11 exp Biomarkers/

12 (biomarker* or bio-marker*).mp.

13 (desmosine or aggrecan or calponin or creatinine kinase or C-reactive protein or elastin or matrix metalloproteinase*).mp.

14 Fibrin Fibrinogen Degradation Products/

15 d-dimer.mp.

16 ADD-RS.mp.

17 or/8-16

18 exp "Sensitivity and Specificity"/ or sensitivity.tw. or specificity.tw. or ((pre-test or pretest) adj probability).tw. or post-test probability.tw. or predictive value$.tw. or likelihood ratio$.tw. or ROC.tw.

19 7 and 17 and 18

| **Databases searched:** | **EMBASE** |
| --- | --- |
| **Platform or provider used:** | **Ovid SP** |
| **Date of coverage:** | **1974 to February 2024** |
| **Search undertaken:** | **February 2024** |

1 acute aortic syndrome/

2 aneurysm, dissecting/ or exp aortic aneurysm/ or dissecting aneurysm/ or acute aortic syndrome/ or aorta dissection/

3 (AAS or (aortic syndrome* and acute)).mp.

4 ((aort* adj3 dissect*) and acute*).mp.

5 ((intramural adj3 h?ematoma*) or (intra-mural adj3 h?ematoma*)).mp.

6 ((aort* adj3 ulcer*) and (atherosclero* or penetrat* or symptom*)).mp.

7 1 or 2 or 3 or 4 or 5 or 6

8 (scor* or biomarker* or D-dimer*).mp.

9 clinical decision rules/ or exp clinical decision support system/

10 clinical decision rule*.mp.

11 exp biological marker/

12 (biomarker* or bio-marker*).mp.

13 (desmosine or aggrecan or calponin or creatinine kinase or C-reactive protein or elastin or matrix metalloproteinase*).mp.

14 exp fibrin degradation product/

15 d-dimer.mp.

16 ADD-RS.mp.

17 or/8-16

18 exp "Sensitivity and Specificity"/ or sensitivity.tw. or specificity.tw. or ((pre-test or pretest) adj probability).tw. or post-test probability.tw. or predictive value$.tw. or likelihood ratio$.tw. or ROC.tw.

19 7 and 17 and 18

| **Databases searched:** | **Cochrane CENTRAL Register of Randomised Controlled Trials & Cochrane Database of Systematic Reviews** |
| --- | --- |
| **Platform or provider used:** | **www.thecochranelibrary.com** |
| **Date of coverage:** | **Inception to February 2024** |
| **Search undertaken:** | **February 2024** |

#1 MeSH descriptor: [Aneurysm, Dissecting] explode all trees

#2 MeSH descriptor: [Aortic Aneurysm] explode all trees

#3 (AAS or (aortic syndrome* and acute)):ti,ab,kw (Word variations have been searched)

#4 ((aort* near/3 dissect*) and acute*):ti,ab,kw (Word variations have been searched)

#5 ((intramural near/3 hematoma*)):ti,ab,kw OR ((intramural near/3 haematoma*)):ti,ab,kw OR ((intra-mural near/3 hematoma*)):ti,ab,kw OR ((intra-mural near/3 haematoma*)):ti,ab,kw (Word variations have been searched)

#6 (aort* near/3 ulcer*):ti,ab,kw AND (atherosclero* or penetrat* or symptom*):ti,ab,kw (Word variations have been searched)

#7 #1 or #2 or #3 or #4 or #5 or #6

#8 ((scor* or biomarker* or D-dimer*)):ti,ab,kw (Word variations have been searched)

#9 MeSH descriptor: [Clinical Decision Rules] explode all trees

#10 MeSH descriptor: [Biomarkers] explode all trees

#11 (clinical decision rule*):ti,ab,kw OR (biomarker* or bio-marker* or biological marker*):ti,ab,kw (Word variations have been searched)

#12 MeSH descriptor: [Fibrin Fibrinogen Degradation Products] explode all trees

#13 (fibrin degradation product*):ti,ab,kw OR (fibrinogen degradation product*):ti,ab,kw (Word variations have been searched)

#14 (desmosine or aggrecan or calponin or "creatinin* kinase*" or "C-reactive protein*" or elastin or "matrix metalloproteinase*"):ti,ab,kw (Word variations have been searched)

#15 (d-dimer or ADD-RS):ti,ab,kw (Word variations have been searched)

#16 #8 or #9 or #10 or #11 or #12 or #13 or #14 or #15

#17 MeSH descriptor: [Sensitivity and Specificity] explode all trees

#18 (sensitivity or specificity or ROC):ti,ab,kw OR ((pre-test or pretest) near/2 probability):ti,ab,kw OR (post-test probability):ti,ab,kw OR (predictive value*):ti,ab,kw OR (likelihood ratio*):ti,ab,kw (Word variations have been searched)

#19 #17 or #18

#20 #7 and #16 and #19
