# Supplementary material for: Diagnostic accuracy of the aortic dissection detection risk score alone or with D-dimer for acute aortic syndromes: Systematic review and meta-analysis
Source: PLoS One. 2024 Jun 21;19(6):e0304401. doi: 10.1371/journal.pone.0304401 (PMC11192411; doi:10.1371/journal.pone.0304401)
Supplement: S4 Appendix — (DOCX) [file pone.0304401.s004.docx]

**S4 Appendix. Statistical models for the meta-analysis**

**Model for multiple thresholds**

The aortic dissection detection risk score (ADD-RS) can take value between zero and three, with multiple thresholds commonly used in practice. In order to accommodate the estimates of sensitivity and specificity at multiple thresholds from each study, we adopted a multinomial meta-analysis model developed by Jones et al. (2019). The model is briefly described as follows.

For each study $i$, we have the number of healthy patients $N_{i}$, the number of diseased patients $P_{i}$, and the number of thresholds $T_{i}$. Test results above a given threshold are considered positive. The number of false positives and true positives at threshold $C_{i,t}$ are denoted by (${FP}_{i,t}, {TP}_{i,t}$), with ${t=1,2,\ldots,T}_{i}$.

Jones et al. (2019) proposed to model the dependency between different threshold values by linking the results from different thresholds using conditional distributions. Specifically, the observed count data (${FP}_{i,t}, {TP}_{i,t}$) at threshold $C_{i,t}$ are modelled using the following formula,

$${FP}_{i,1}\sim Binomial \left( N_{i}, {FPR}_{i,1} \right)$$

$${FP}_{i,t}|{FP}_{i,t-1}\sim Binomial \left( \mathrm{FP}_{i,t-1}, {FPR}_{i,t}/{FPR}_{i,t-1} \right)$$

$${TP}_{i,1}\sim Binomial \left( P_{i}, {TPR}_{i,1} \right)$$

${TP}_{i,t}|{TP}_{i,t-1}\sim Binomial \left( \mathrm{TP}_{i,t-1}, {TPR}_{i,t}/{TPR}_{i,t-1} \right)$.

Here, ${FPR}_{i,t}$ and ${TPR}_{i,t}$ represent the false positive rates and true positive rates of study $i$ at threshold $t$ respectively.

In the multinomial meta-analysis model, ${FPR}_{i,t}$ and ${TPR}_{i,t}$ depend on the individual mean and scale parameters $\mu_{Ai}, \mu_{Bi}$, ${log(\sigma}_{Ai}), {log(\sigma}_{Bi})$, and the specific threshold $C_{i,t}$ by the following specification,

$$logit\left( {FPR}_{i,t} \right)=\frac{\mu_{Ai}-g(C_{i,t})}{\sigma_{Ai}}$$

$$logit\left( {TPR}_{i,t} \right)=\frac{\mu_{Bi}-g(C_{i,t})}{\sigma_{Bi}}$$

For the transformation $g()$, we use a natural logarithm, $g()=log()$ for computational convenience. More details about a flexible form of transformation can be found in Jones et al. (2019).

The individual parameters $\mu_{Ai}$, $\mu_{Bi}$, ${log(\sigma}_{Ai})$, ${log(\sigma}_{Bi})$ are assumed to be normally distributed with mean parameters $m_{A}, m_{B}, s_{A},s_{B}$ and a four-dimensional variance-covariance matrix $\Sigma$,

$\left( \begin{aligned} \mu_{Ai} \\ \mu_{Bi} \\ {log(\sigma}_{Ai}) \\ {log(\sigma}_{Bi}) \end{aligned} \right)$ ~ MVN$\left( \left( \begin{aligned} m_{A} \\ m_{B} \\ s_{A} \\ s_{B} \end{aligned} \right), \Sigma\right)$.

Correlations are generally expected across the four sets of random effects $\mu_{Ai}$, $\mu_{Bi}$, ${log(\sigma}_{Ai})$, ${log(\sigma}_{Bi})$ and the correlation matrix can be pre-specified accordingly. Different correlation structures are described in Jones et al. (2019), including a full correlation matrix which allows for all possible between-study correlations, a structured correlation matrix and an independence model that assumes independence of the random effects.

**Model for a single threshold**

A bivariate model is used for analysing sensitivity and specificity jointly by allowing for correlation between them. In particular, the observed number of true positives in study 𝑖, 𝑇𝑃_𝑖_, is assumed to be binomially distributed with parameter, $\pi_{Ai}$, representing the study-specific sensitivity given the total number of positives on the reference test such that:

$${TP}_{i}\sim Binomial \left( \left( {TP}_{i}+{FN}_{i} \right), \pi_{Ai} \right).$$

Similarly, the observed number of true negatives in study 𝑖, 𝑇𝑁_𝑖_, is assumed to be binomially distributed with parameter, $\pi_{Bi},$representing the study-specific specificity given the total number of negatives on the reference test such that:

$${TN}_{i}\sim Binomial \left( {\left( {TN}_{i}+{FP}_{i} \right), \pi}_{Bi} \right).$$

The probabilities are transformed to a logit scale:

$$\mu_{Ai}=\mathrm{logit}\left( \pi_{Ai} \right)$$

$$\mu_{Bi}=\mathrm{logit}\left( \pi_{Bi} \right)$$

The study-specific sensitivity and specificity are jointly modelled using a bivariate normal distribution:

$$\left( \begin{matrix} \mu_{Ai} \\ \mu_{Bi} \end{matrix} \right)\sim N\left( \begin{matrix} m_{A} \\ m_{B} \end{matrix},\Sigma_{AB} \right)$$

$$\Sigma_{AB}=\left( \begin{matrix} \sigma_{A}^{2} & \sigma_{AB} \\ \sigma_{AB} & \sigma_{B}^{2} \end{matrix} \right)$$

where $m_{A}$ and $m_{B}$ represent the population mean for logit sensitivity and specificity; $\sigma_{A}^{2}$represents the variability in the logit sensitivity between studies, $\sigma_{B}^{2}$ represents the variability in the logit specificity between studies and $\sigma_{AB}$represents the covariance of the logit sensitivity and logit specificity. Specifically, we rewrite the covariance as $\sigma_{AB}=\rho\sigma_{A}\sigma_{B}$, with $\rho$ being the correlation coefficient and we place prior distributions over $\rho{, \sigma}_{A}, \sigma_{B}$ in the Bayesian analysis.

**Prior specification**

Prior distributions are required for the hyperparameters. For the ADD-RS main analysis with 12 studies, we adopted a reference prior for the hyperparameters as recommended by Jones et al. (2019). Specifically, a normal distribution $N (0, 100)$ is used as the prior for the mean parameters $m_{A}, m_{B}, s_{A},s_{B}$, and a uniform distribution $U\left( 0, 5 \right)$ is used as the prior for the standard deviation of the random effects $\mu_{Ai}$, $\mu_{Bi}$, ${log(\sigma}_{Ai})$, ${log(\sigma}_{Bi})$. For the corelation structure, we explored using an independent correlation matrix with the four sets of parameters assumed to be independent of each other, and a structured correlation matrix with a uniform distribution $U\left( -1,1 \right)$ used as prior over the correlation coefficient.

After a total 1,000,000 iterations with a burn-in of 100,000 and thinning of 10, the DIC is 239.51 with an independent reference prior and the DIC is 239.54 with the structured reference prior. Including additional parameters for between-study correlations did not improve the model fit according to the DIC. Therefore the simpler independence model was used in the ADD-RS main analysis. The point estimates of the pooled sensitivity and the specificity of the two different priors are quite similar, and the credible intervals and the prediction intervals are slightly wider for the structure prior compared to the independent prior, as presented in Table S4.

For the ADD-RS sensitivity analysis, ADD-RS with D-Dimer analysis, and Canadian guideline analysis, we used informative priors for the standard deviation as there are only limited number of studies available for analysis. The use of informative prior can help to incorporate external information into the model so that a better estimation can be obtained for the between-study correlation matrix. An informative prior is obtained by fitting a parametric distribution to the posterior samples from the ADD-RS main analysis. We found that the gamma distribution has a good fit to the posterior samples and a good convergence, compared to the log-normal distribution and the normal distribution. Specifically, we selected gamma distributions $\Gamma(13.2, 44.7)$, $\Gamma(7.4, 38.3)$, $\Gamma(9.3, 38.1)$, $\Gamma(4.4, 15.2)$ as the prior distribution over the standard deviation.

We also explored using a reference prior with an independent correlation structure for the ADD-RS sensitivity analysis and ADD-RS with D-Dimer analysis, which means that a normal distribution $N (0, 100)$ is used as the prior distribution over the mean parameters $m_{A}, m_{B}, s_{A},s_{B}$, and a uniform distribution $U(0, 5)$ is used as the prior over the standard deviation of the random effects. Results of the ADD-RS sensitivity analysis, ADD-RS with D-Dimer analysis, and Canadian guideline analysis with different priors are presented in Table S4. The point estimates of the pooled sensitivity and the specificity of the two different priors are quite similar. The prediction intervals and the credible intervals are wider with the reference prior compared to the informative prior.

**Table S4: Pooled estimates for each analysis**

| **Strategy** | **Threshold** | **Prior specification** | **Sensitivity (%) (95% CrI)**  **[95% PrI]** | **Specificity (%)**  **(95% CrI)**  **[95% PrI]** |
| --- | --- | --- | --- | --- |
| ADD-RS main analysis | ADD-RS>0 | **Independent reference prior**  **(preferred)** | **94.6**  **(90, 97.5)**  **[72.6, 99.7]** | **34.7**  **(20.7, 51.2)**  **[3.3, 86.9]** |
|  |  | Structured reference prior | 94.6  (89.8, 97.6)  [69.7, 99.8] | 34.6  (19.7,52.8)  [2.5, 89.6] |
|  | ADD-RS>1 | **Independent reference prior**  **(preferred)** | **43.4**  **(31.2, 57.1)**  **[9.9, 83.3]** | **89.3**  **(80.4, 94.8)**  **[41.9, 99.5]** |
|  |  | Structured reference prior | 43.5  (30.5, 58.2)  [8.5, 85.8] | 89.3  (79.2, 95.1)  [36.2, 99.6] |
| ADD-RS with D-Dimer analysis | ADD-RS>0 or D-dimer>500 | **Informative gamma prior**  **(preferred)** | **99.8**  **(98.7, 100)**  **[96.1, 100]** | **21.8**  **(12.1, 32.6)**  **[2.6, 50.7]** |
|  |  | Reference prior | 99.7  (96.9,100)  [74.1, 100] | 22.1  (6.6, 39.9)  [0.1, 69.1] |
|  | ADD-RS>or D-dimer>500 | **Informative gamma prior**  **(preferred)** | **98.3**  **(94.9, 99.5) [86.4, 100]** | **51.4**  **(38.7, 64.1)**  **[18.5, 83.5]** |
|  |  | Reference prior | 98.3  (89.2, 99.8)  [44, 100] | 51.4  (31.6, 71.8)  [4.6, 96.5] |
|  | ADD RS>1, ADD RS=1 and D-dimer>500 | **Informative gamma prior**  **(preferred)** | **93.1**  **(87.1, 96.3) [74.1, 98.3]** | **67.1**  **(54.4, 77.7)**  **[33.4, 89.3]** |
|  |  | Reference prior | 93.1  (81.8, 97)  [50, 99.3] | 67.1  (47.5, 82.3)  [19.2, 94.7] |
| ADD-RS sensitivity analysis | ADD-RS>0 | **Informative gamma prior**  **(preferred)** | **95.1**  **(88.5, 98.4)**  **[72.9, 99.8]** | **38**  **(20.1, 59.1)**  **[4.5, 86.8]** |
|  |  | Reference prior | 95.1  (86.1, 98.8)  [64.4, 99.9] | 38  (20.1, 58.7)  [4, 86.7] |
|  | ADD-RS>1 | **Informative gamma prior**  **(preferred)** | **41.6**  **(24.8, 59.1)**  **[8.1, 82.5]** | **91.7**  **(81.7, 97)**  **[53.7, 99.6]** |
|  |  | Reference prior | 41.7  (21.9, 61.8)  [4.9, 87.7] | 91.7  (81.1, 97.4)  [54.5, 99.8] |

ADD-RS: Aortic Dissection Detection Risk Score; CrI Credible Interval; PrI: Prediction Interval. Results with preferred priors are in bold.
