# Supplementary material for: Diagnostic accuracy of the aortic dissection detection risk score alone or with D-dimer for acute aortic syndromes: Systematic review and meta-analysis
Source: PLoS One. 2024 Jun 21;19(6):e0304401. doi: 10.1371/journal.pone.0304401 (PMC11192411; doi:10.1371/journal.pone.0304401)
Supplement: S5 Appendix — (DOCX) [file pone.0304401.s005.docx]

**S5 Appendix. List of excluded studies with rationale**

|  | **Authors, year** | **Reason for exclusion** |
| --- | --- | --- |
|  | Ahmadi et al., 2018^1^ | Review (no primary data) |
|  | Akutsu et al., 2005^2^ | Index test - not relevant |
|  | Baez and Cochon, 2017^3^ | Review (no primary data) |
|  | Barry and Coughlan, 2019^4^ (abstract) | Index test - not relevant (abstract only) |
|  | Cakir et al., 2021^5^ | Population - not suspected AAS |
|  | Chen et al., 2023^6^ | Review (no primary data) |
|  | Cheng et al., 2020^7^ | Population - not suspected AAS |
|  | Derksen et al., 2018^8^ (abstract) | Index test - not relevant (abstract only) |
|  | Dong et al., 2017a^9^ | Population - not suspected AAS |
|  | Dong et al., 2017b^10^ | Population - not suspected AAS |
|  | Duceau et al., 2020^11^ | Index test - not relevant |
|  | Eggebrecht et al., 2004^12^ | Population - not suspected AAS |
|  | Ersel et al., 2010^13^ | Index test - not relevant (abstract only) |
|  | Fan et al., 2010^14^ | Index test - not relevant (abstract only) |
|  | Fletcher et al., 2021^15^ | Population - not suspected AAS |
|  | Forrer et al., 2021^16^ | Population - not suspected AAS |
|  | Gawinecka et al., 2021^17^ (abstract) | Abstract of a full text study (Forrer et al., 2021^16^) |
|  | Giachino et al., 2013^18^ | Index test - not relevant |
|  | Giachino et al., 2014^19^ (abstract) | Population - not suspected AAS (abstract only) |
|  | Goliopoulou et al., 2022^20^ (abstract) | Population - not suspected AAS (abstract only) |
|  | Gorla et al.,2017b^21^ | Case-control (unselected controls with suspected AAS) |
|  | Guo et al., 2013^22^ | Population - not suspected AAS |
|  | Hagiwara et al., 2010^23^ (abstract) | Population - not suspected AAS (abstract only) |
|  | Hagiwara et al., 2013^24^ | Population - not suspected AAS |
|  | Han et al., 2021^25^ | Population - not suspected AAS |
|  | Hashemi et al., 2021^26^ (abstract) | Abstract of included full text study (Zitek et al 2022) |
|  | Hazui et al., 2005^27^ | Population - not suspected AAS |
|  | Inaba et al., 2018^28^ (abstract) | Index test - not relevant (abstract only) |
|  | Jiang et al., 2022^29^ | Population - not suspected AAS |
|  | Kaito et al., 2022^30^ | Population - not suspected AAS |
|  | Khan et al., 2023^31^ (abstract) | Outcome – no useable data |
|  | Konig et al., 2021^32^ | Population - not suspected AAS |
|  | Konig et al., 2021^33^ (abstract) | Abstract of a full text study (Konig et al., 2021^32^) |
|  | Lee et al., 2022^34^ | Index test - not relevant |
|  | Levcik et al., 2013^35^ | Index test - not relevant |
|  | Li et al., 2010^36^ (abstract) | Index test - not relevant (abstract only) |
|  | Li et al., 2017^37^ | Index test - not relevant |
|  | Li et al., 2018^38^ | Population - not suspected AAS |
|  | Li et al., 2022^39^ | Population - not suspected AAS |
|  | Lian et al., 2023^40^ | Index test - not relevant |
|  | Liu et al., 2018^41^ | Population - not suspected AAS |
|  | Liu et al., 2022^42^ | Index test - not relevant |
|  | Lovy et al., 2013^43^ | Index test - not relevant |
|  | Lu et al., 2022^44^ | Population - not suspected AAS |
|  | Meng et al., 2019^45^ | Index test - not relevant |
|  | Morello et al., 2016^46^ | Index test - not relevant |
|  | Morello et al., 2017^47^ | Index test - not relevant |
|  | Morello et al., 2017^48^ (abstract) | Abstract of included full text study (Nazerian et al 2018) |
|  | Morello et al., 2018^49^ | Data overlaps with Nazerian 2018 |
|  | Morello et al., 2020^50^ | Data overlaps with Morello 2021 |
|  | Morello et al., 2023^51^ | All patients have AAS |
|  | Nazerian et al., 2013^52^ (abstract) | Abstract of included full text study (Nazerian et al 2014b) |
|  | Nazerian et al., 2018^53^ (abstract) | Abstract of included full text study (Nazerian et al 2018) |
|  | Ohle et al., 2017^54^ (abstract) | Duplicate |
|  | Ohle et al., 2018a^55^ (abstract) | Abstract of included full text study (Ohle et al 2018) |
|  | Ohle et al., 2018b^56^ | Case-control (unselected controls with suspected AAS) |
|  | Ohle et al., 2019^57^ (abstract) | Index test - not relevant (abstract only) |
|  | Ohle et al., 2023^58^ | Case-control (unselected controls with suspected AAS) |
|  | Ohlmann et al., 2006^59^ | Case-control (unselected controls with suspected AAS) |
|  | Okazaki et al., 2014^60^ | Population - not suspected AAS |
|  | Paige et al., 2020^61^ (abstract) | Duplicate |
|  | Pan et al., 2021^62^ | Population - not suspected AAS |
|  | Peng et al., 2015^63^ | Index test - not relevant |
|  | Qiming et al., 2010^64^ (abstract) | Index test - not relevant (abstract only) |
|  | Reeps et al., 2010^65^ | All patients have AAS |
|  | Sakamoto et al., 2011^66^ | Population - not suspected AAS |
|  | Sakamoto et al., 2016^67^ | Population - not suspected AAS |
|  | Sbarouni et al., 2007^68^ | Population - not suspected AAS |
|  | Sbarouni et al., 2015^69^ | Population - not suspected AAS |
|  | Sbarouni et al., 2018^70^ | Population - not suspected AAS |
|  | Shao et al., 2014^71^ | Case-control (unselected controls with suspected AAS) |
|  | Shinohara et al., 2003^72^ | Population - not suspected AAS |
|  | Shirakabe et al., 2008^73^ | Population - not suspected AAS |
|  | Song et al., 2022^74^ | Population - not suspected AAS |
|  | Song et al., 2023^75^ | Case-control (unselected controls with suspected AAS) |
|  | Spinner et al., 2006^76^ (abstract) | Index test - not relevant |
|  | Stanojlovic et al., 2013^77^ (abstract) | Index test - not relevant |
|  | Suzuki et al., 1996^78^ | Population - not suspected AAS |
|  | Suzuki et al., 2008^79^ | Index test - not relevant |
|  | Suzuki et al., 2009^80^ | Index test - not relevant |
|  | Suzuki et al., 2011^81^ | Population - not suspected AAS |
|  | Tokuda et al., 2016^82^ | Population - not suspected AAS (abstract only) |
|  | Tokuda et al., 2018^83^ | Population - not suspected AAS |
|  | von Kodolitsch et al., 2000^84^ | Index test - not relevant |
|  | Wagner et al., 2002^85^ | Population - not suspected AAS |
|  | Wang et al., 2018^86^ | Population - not suspected AAS |
|  | Wang et al., 2018^87^ | Index test - not relevant |
|  | Weber et al., 2003^88^ | Case-control (unselected controls with suspected AAS) |
|  | Wei et al., 2012^89^ (abstract) | All patients have AAS |
|  | Wiegand et al., 2007^90^ | All patients have AAS |
|  | Wilson et al., 2016^91^ (abstract) | Index test - not relevant (abstract only) |
|  | Xiao et al., 2016^92^ | Case-control (unselected controls with suspected AAS) |
|  | Xu et al., 2017^93^ | Population - not suspected AAS |
|  | Xu et al., 2022^94^ | Population - not suspected AAS |
|  | Xue et al., 2007^95^ | Index test - not relevant |
|  | Yoshimuta et al., 2015^96^ | Population - not suspected AAS |
|  | Yuan et al., 2011^97^ | Population - not suspected AAS |
|  | Zeng et al., 2020^98^ | Population - not suspected AAS |
|  | Zhang et al., 2023^99^ | Index test - not relevant |
|  | Zhang et al., 2023^100^ | Case-control (unselected controls with suspected AAS) |
|  | Zhang et al., 2023^101^ | Duplicate |
|  | Zhao et al., 2020^102^ | Population - not suspected AAS |
|  | Zitek et al., 2022^103^ | Case-control (unselected controls with suspected AAS) |
|  | Zheng et al.,  2012^104^ (abstract) | Population - not suspected AAS (abstract only) |

**REFERENCES (Appendix S3)**

1. Ahmadi I, Qavam SM, Sayehmiri K, et al. Investigating the efficiency of D-Dimer test in diagnosis of aortic dissection: A systematic study and meta-analysis. *International Cardiovascular Research Journal* 2018;12(4):142-47.

2. Akutsu K, Sato N, Yamamoto T, et al. A rapid bedside D-dimer assay (cardiac D-dimer) for screening of clinically suspected acute aortic dissection. *Circulation journal : official journal of the Japanese Circulation Society* 2005;69(4):397-403.

3. Baez AA, Cochon L. Improved rule-out diagnostic gain with a combined aortic dissection detection risk score and D-dimer Bayesian decision support scheme. *Journal of critical care* 2017;37:56-59. doi: <https://dx.doi.org/10.1016/j.jcrc.2016.08.007>

4. Barry I, Coughlan F. Computed tomography thoracic angiography referred from a tertiary ED: An audit to determine if this test is being utilised optimally in the investigation of acute aortic dissection. *Journal of Medical Imaging and Radiation Oncology* 2019;63(Supplement 1):154-55.

5. Cakir A, Payza U, Aksun S, et al. Validity of signal peptide-cub-egf domain-containing protein-1 (Scube-1) in the diagnosis of aortic dissection. *Signa Vitae* 2021;17(1):112-16.

6. Chen H, Li Y, Li Z, et al. Diagnostic biomarkers and aortic dissection: a systematic review and meta-analysis. *BMC cardiovascular disorders* 2023;23(1):497. doi: <https://dx.doi.org/10.1186/s12872-023-03448-9>

7. Cheng N, Wang H, Zhang W, et al. Comparative Proteomic Investigation of Plasma Reveals Novel Potential Biomarker Groups for Acute Aortic Dissection. *Disease markers* 2020;2020:4785068. doi: <https://dx.doi.org/10.1155/2020/4785068>

8. Derksen B, Glober N, Darocki M, et al. Is the highly sensitive HemosIL D-dimer a valuable screening tool to rule out aortic dissection? *Academic Emergency Medicine* 2018;25(Supplement 1):S197-S98.

9. Dong J, Bao J, Feng R, et al. Circulating microRNAs: a novel potential biomarker for diagnosing acute aortic dissection. *Scientific reports* 2017;7(1):12784. doi: <https://dx.doi.org/10.1038/s41598-017-13104-w>

10. Dong J, Duan X, Feng R, et al. Diagnostic implication of fibrin degradation products and D-dimer in aortic dissection. *Scientific reports* 2017;7:43957. doi: <https://dx.doi.org/10.1038/srep43957>

11. Duceau B, Alsac JM, Bellenfant F, et al. Prehospital triage of acute aortic syndrome using a machine learning algorithm. *The British journal of surgery* 2020;107(8):995-1003. doi: <https://dx.doi.org/10.1002/bjs.11442>

12. Eggebrecht H, Naber CK, Bruch C, et al. Value of plasma fibrin D-dimers for detection of acute aortic dissection. *Journal of the American College of Cardiology* 2004;44(4):804-9.

13. Ersel M, Aksay E, Kiyan S, et al. Can D-dimer testing help emergency department physicians to detect acute aortic dissections? *Anadolu kardiyoloji dergisi : AKD = the Anatolian journal of cardiology* 2010;10(5):434-9. doi: <https://dx.doi.org/10.5152/akd.2010.142>

14. Fan Q-k, Wang W-w, Zhang Z-l, et al. Evaluation of D-dimer in the diagnosis of suspected aortic dissection. *Clinical chemistry and laboratory medicine* 2010;48(12):1733-7. doi: <https://dx.doi.org/10.1515/CCLM.2010.337>

15. Fletcher A, Syed M, Iskander Z, et al. Plasma desmosine as a biomarker in acute aortic syndrome. *European Heart Journal* 2021;42(Supplement_1):ehab724. 2011.

16. Forrer A, Schoenrath F, Torzewski M, et al. Novel Blood Biomarkers for a Diagnostic Workup of Acute Aortic Dissection. *Diagnostics (Basel, Switzerland)* 2021;11(4) doi: <https://dx.doi.org/10.3390/diagnostics11040615>

17. Gawinecka J, Forrer A, Schonrath F, et al. Novel biomarkers in diagnostic workup of acute aortic dissection. *Atherosclerosis* 2021;331:e248.

18. Giachino F, Loiacono M, Lucchiari M, et al. Rule out of acute aortic dissection with plasma matrix metalloproteinase 8 in the emergency department. *Critical care (London, England)* 2013;17(1):R33. doi: <https://dx.doi.org/10.1186/cc12536>

19. Giachino F, Bono A, Castelli M, et al. Diagnostic performance of D-dimer in patients with history of aortic dissection. *Giornale Italiano di Cardiologia* 2014;2):e161.

20. Goliopoulou A, Oikonomou E, Antonopoulos A, et al. Expression of Tissue microRNAs in Ascending Aortic Aneurysms and Dissections. *Angiology* 2022:33197221098295.

21. Gorla R, Erbel R, Kahlert P, et al. Diagnostic role and prognostic implications of D-dimer in different classes of acute aortic syndromes. *European heart journal Acute cardiovascular care* 2017;6(5):379-88. doi: <https://dx.doi.org/10.1177/2048872615594500>

22. Guo Z-g, Ma Q-b, Zheng Y-a, et al. [The value of D-dimer for etiological diagnosis of mortal chest pain: an analysis of 438 cases]. *Zhonghua wei zhong bing ji jiu yi xue* 2013;25(11):655-9. doi: <https://dx.doi.org/10.3760/cma.j.issn.2095-4352.2013.11.005>

23. Hagiwara A, Sakamoto D, Sasaki R, et al. Diagnosis of acute aortic dissection using a fibrinolytic marker. *Critical Care Medicine* 2010;12):A178.

24. Hagiwara A, Shimbo T, Kimira A, et al. Using fibrin degradation products level to facilitate diagnostic evaluation of potential acute aortic dissection. *Journal of thrombosis and thrombolysis* 2013;35(1):15-22. doi: <https://dx.doi.org/10.1007/s11239-012-0779-6>

25. Han C, Liu Q, Li Y, et al. S100A1 as a potential biomarker for the diagnosis of patients with acute aortic dissection. *The Journal of international medical research* 2021;49(4):3000605211004512. doi: <https://dx.doi.org/10.1177/03000605211004512>

26. Hashemi M, Zagroba S, Zitek T. 299 The Diagnostic Value of D-dimer Levels for the Exclusion of Aortic Dissection. *Annals of Emergency Medicine* 2021;78(4 Supplement):S121-S22.

27. Hazui H, Fukumoto H, Negoro N, et al. Simple and useful tests for discriminating between acute aortic dissection of the ascending aorta and acute myocardial infarction in the emergency setting. *Circulation journal : official journal of the Japanese Circulation Society* 2005;69(6):677-82.

28. Inaba H, Yamashita A, Yoshita Y, et al. Do emergency medical service systems properly asssess the risk of acute aortic syndome and transport the patients to the right hospitals? Impact of ems assessment and transportation in outcomes. *Cardiology (Switzerland)* 2018;140(Supplement 1):299.

29. Jiang Y, Tang X, Wang Y, et al. Serum Oxylipin Profiles Identify Potential Biomarkers in Patients with Acute Aortic Dissection. *Metabolites* 2022;12(7) (no pagination)

30. Kaito D, Yamamoto R, Nakama R, et al. D-dimer for screening of aortic dissection in patients with ST-elevation myocardial infarction. *American Journal of Emergency Medicine* 2022;59:146-51.

31. Khan A, Masoura C, Mitra A, et al. Accuracy of aortic dissection detection risk score and D-dimer to rule out acute aortic syndromes in the emergency department. *Critical Care* 2023;27(Supplement 1) doi: <https://dx.doi.org/10.1186/s13054-023-04377-x>

32. Konig KC, Lahm H, Dresen M, et al. Aggrecan: a new biomarker for acute type A aortic dissection. *Scientific reports* 2021;11(1):10371. doi: <https://dx.doi.org/10.1038/s41598-021-89653-y>

33. Konig C, Lahm H, DreBen M, et al. Aggrecan: A new biomarker for acute thoracic aortic dissection. *Thoracic and Cardiovascular Surgeon Conference: 50th Annual Meeting of the German Society for Thoracic and Cardiovascular Surgery, DGTHG* 2021;69(SUPPL 1)

34. Lee D, Kim YW, Kim TY, et al. Age-Adjusted D-Dimer in Ruling Out Acute Aortic Syndrome. *Emergency medicine international* 2022;2022:6864756. doi: <https://dx.doi.org/10.1155/2022/6864756>

35. Levcik M, Kettner J, Jabor A, et al. Utility of plasma D-dimer levels in the diagnosis of acute aortic dissection. *Cor et Vasa* 2013;55(6):e510-e14. doi: <https://dx.doi.org/10.1016/j.crvasa.2013.04.009>

36. Li W, Fan X, Xu J, et al. The value of D-dimer in acute aortic dissection: The experience of China. *Atherosclerosis Supplements* 2010;11(2):197.

37. Li W, Huang B, Tian L, et al. Admission D-dimer testing for differentiating acute aortic dissection from other causes of acute chest pain. *Archives of medical science : AMS* 2017;13(3):591-96. doi: <https://dx.doi.org/10.5114/aoms.2017.67280>

38. Li T, Jiang B, Li X, et al. Serum matrix metalloproteinase-9 is a valuable biomarker for identification of abdominal and thoracic aortic aneurysm: a case-control study. *BMC cardiovascular disorders* 2018;18(1):202. doi: <https://dx.doi.org/10.1186/s12872-018-0931-0>

39. Li T, Zhou Y, Li D, et al. The role of genome-scale leukocyte long noncoding RNA in identifying acute aortic dissection. *Signa Vitae* 2022;18(3):101-10.

40. Lian R, Zhang T, Liu J, et al. Routine Use of a Pocket-Sized Handheld Echoscopic Device Plus a Biomarker by Emergency Medicine Residents with an Early Screening Algorithm for Suspected Type A Acute Aortic Syndrome. *Journal of Clinical Medicine* 2023;12(4):1346. doi: <https://dx.doi.org/10.3390/jcm12041346>

41. Liu X, Zheng X, Su X, et al. Plasma Resistin Levels in Patients with Acute Aortic Dissection: A Propensity Score-Matched Observational Case-Control Study. *Medical science monitor : international medical journal of experimental and clinical research* 2018;24:6431-37. doi: <https://dx.doi.org/10.12659/MSM.909469>

42. Liu W-T, Lin C-S, Tsao T-P, et al. A Deep-Learning Algorithm-Enhanced System Integrating Electrocardiograms and Chest X-rays for Diagnosing Aortic Dissection. *The Canadian journal of cardiology* 2022;38(2):160-68. doi: <https://dx.doi.org/10.1016/j.cjca.2021.09.028>

43. Lovy AJ, Bellin E, Levsky JM, et al. Preliminary development of a clinical decision rule for acute aortic syndromes. *The American journal of emergency medicine* 2013;31(11):1546-50. doi: <https://dx.doi.org/10.1016/j.ajem.2013.06.005>

44. Lu P, Feng X, Li R, et al. A Novel Serum Biomarker Model to Discriminate Aortic Dissection from Coronary Artery Disease. *Disease Markers* 2022;2022:9716424.

45. Meng J, Mellnick VM, Monteiro S, et al. Acute Aortic Syndrome: Yield of Computed Tomography Angiography in Patients With Acute Chest Pain. *Canadian Association of Radiologists Journal* 2019;70(1):23-28.

46. Morello F, Ravetti A, Nazerian P, et al. Plasma Lactate Dehydrogenase Levels Predict Mortality in Acute Aortic Syndromes: A Diagnostic Accuracy and Observational Outcome Study. *Medicine* 2016;95(6):e2776. doi: <https://dx.doi.org/10.1097/MD.0000000000002776>

47. Morello F, Cavalot G, Giachino F, et al. White blood cell and platelet count as adjuncts to standard clinical evaluation for risk assessment in patients at low probability of acute aortic syndrome. *European heart journal Acute cardiovascular care* 2017;6(5):389-95. doi: <https://dx.doi.org/10.1177/2048872615600097>

48. Morello F, Nazerian P, Mueller C, et al. Rule-out of acute aortic syndrome by integration of the aortic dissection detection risk score plus d-dimer: Preliminary data from the ADvISED prospective multicenter study. *European Heart Journal* 2017;38(Supplement 1):1264.

49. Morello F, Oddi M, Cavalot G, et al. Prospective diagnostic and prognostic study of copeptin in suspected acute aortic syndromes. *Scientific reports* 2018;8(1):16713. doi: <https://dx.doi.org/10.1038/s41598-018-35016-z>

50. Morello F, Bartalucci A, Bironzo M, et al. Prospective diagnostic accuracy study of plasma soluble ST2 for diagnosis of acute aortic syndromes. *Scientific reports* 2020;10(1):3103. doi: <https://dx.doi.org/10.1038/s41598-020-59884-6>

51. Morello F, Santoro M, Giachino F, et al. Pre-Test Probability Assessment and d-Dimer Based Evaluation in Patients with Previous Acute Aortic Syndrome. *Medicina (Kaunas, Lithuania)* 2023;59(3) doi: <https://dx.doi.org/10.3390/medicina59030548>

52. Nazerian P, Morello F, Vanni S, et al. Combined use of a standardized risk score and d-dimer to rule out acute aortic dissection in the emergency department. *European Heart Journal* 2013;1):1113-14.

53. Nazerian P, Vanni S, Mueller C, et al. Diagnostic performance of enlarged mediastinum on chest X ray in patients with suspected acute aortic dissection. the ADVISED-CXR multicenter prospective study. *European Heart Journal: Acute Cardiovascular Care* 2018;7(1 Supplement 1):338.

54. Ohle R, Um SW, Wells GA, et al. High-risk clinical features for acute aortic syndrome. *Academic Emergency Medicine* 2017;24(Supplement 1):S88.

55. Ohle R, McIsaac S, Perry JJ. A RAPID bedside approach to ruling out acute aortic dissection. *Canadian Journal of Emergency Medicine* 2018;20(Supplement 1):S17-S18.

56. Ohle R, Um J, Anjum O, et al. High Risk Clinical Features for Acute Aortic Dissection: A Case-Control Study. *Academic Emergency Medicine* 2018;25(4):378-87.

57. Ohle R, Fortino N, Montpellier O, et al. Prospective pilot implementation of a clinical decision aid for acute aortic syndrome. *Canadian Journal of Emergency Medicine* 2019;21(Supplement 1):S98.

58. Ohle R, McIsaac S, Van Drusen M, et al. Evaluation of the Canadian Clinical Practice Guidelines Risk Prediction Tool for Acute Aortic Syndrome: The RIPP Score. *Emergency Medicine International* 2023;2023:6636800. doi: <https://dx.doi.org/10.1155/2023/6636800>

59. Ohlmann P, Faure A, Morel O, et al. Diagnostic and prognostic value of circulating D-Dimers in patients with acute aortic dissection. *Critical care medicine* 2006;34(5):1358-64.

60. Okazaki T, Yamamoto Y, Yoda K, et al. The ratio of D-dimer to brain natriuretic peptide may help to differentiate between cerebral infarction with and without acute aortic dissection. *Journal of the neurological sciences* 2014;340(1-2):133-8. doi: <https://dx.doi.org/10.1016/j.jns.2014.03.011>

61. Paige B, Maeng A, Savage D, et al. Validation of the Canadian clinical practice guideline clinical decision aid for acute aortic syndrome. *Canadian Journal of Emergency Medicine* 2020;22(Supplement 1):S43-S44.

62. Pan X, Zhou Y, Yang G, et al. Lysophosphatidic Acid May Be a Novel Biomarker for Early Acute Aortic Dissection. *Frontiers in surgery* 2021;8:789992. doi: <https://dx.doi.org/10.3389/fsurg.2021.789992>

63. Peng W, Peng Z, Chai X, et al. Potential biomarkers for early diagnosis of acute aortic dissection. *Heart & lung : the journal of critical care* 2015;44(3):205-8. doi: <https://dx.doi.org/10.1016/j.hrtlng.2015.01.006>

64. Qiming L, Ming T, Shenghua Z, et al. Analysis on early diagnosis grading model of acute aortic dissection. *Heart* 2010;3):A187.

65. Reeps C, Pelisek J, Bundschuh RA, et al. Imaging of acute and chronic aortic dissection by 18F-FDG PET/CT. *Journal of Nuclear Medicine* 2010;51(5):686-91.

66. Sakamoto K, Yamamoto Y, Okamatsu H, et al. D-dimer is helpful for differentiating acute aortic dissection and acute pulmonary embolism from acute myocardial infarction. *Hellenic journal of cardiology : HJC = Hellenike kardiologike epitheorese* 2011;52(2):123-7.

67. Sakamoto Y, Koga M, Ohara T, et al. Frequency and Detection of Stanford Type A Aortic Dissection in Hyperacute Stroke Management. *Cerebrovascular diseases (Basel, Switzerland)* 2016;42(1-2):110-6. doi: <https://dx.doi.org/10.1159/000445528>

68. Sbarouni E, Georgiadou P, Marathias A, et al. D-dimer and BNP levels in acute aortic dissection. *International journal of cardiology* 2007;122(2):170-2.

69. Sbarouni E, Georgiadou P, Analitis A, et al. High neutrophil to lymphocyte ratio in type A acute aortic dissection facilitates diagnosis and predicts worse outcome. *Expert review of molecular diagnostics* 2015;15(7):965-70. doi: <https://dx.doi.org/10.1586/14737159.2015.1042367>

70. Sbarouni E, Georgiadou P, Kosmas E, et al. Platelet to lymphocyte ratio in acute aortic dissection. *Journal of clinical laboratory analysis* 2018;32(7):e22447. doi: <https://dx.doi.org/10.1002/jcla.22447>

71. Shao N, Xia S, Wang J, et al. The role of D-dimers in the diagnosis of acute aortic dissection. *Molecular biology reports* 2014;41(10):6397-403. doi: <https://dx.doi.org/10.1007/s11033-014-3520-z>

72. Shinohara T, Suzuki K, Okada M, et al. Soluble elastin fragments in serum are elevated in acute aortic dissection. *Arteriosclerosis, Thrombosis, and Vascular Biology* 2003;23(10):1839-44.

73. Shirakabe A, Hata N, Yokoyama S, et al. Diagnostic score to differentiate acute aortic dissection in the emergency room. *Circulation journal : official journal of the Japanese Circulation Society* 2008;72(6):986-90.

74. Song R, Xu N, Luo L, et al. Diagnostic Value of Aortic Dissection Risk Score, Coagulation Function, and Laboratory Indexes in Acute Aortic Dissection. *BioMed Research International* 2022;2022:7447230.

75. Song DH, Choi JH, Lee JY. Predicting acute aortic syndrome using aortic dissection detection risk score, D-dimer, and X-ray. *Heliyon* 2023;9(10):e20578. doi: <https://dx.doi.org/10.1016/j.heliyon.2023.e20578>

76. Spinner T, Spes C, Mudra H. Elevated d-dimer at acute chest pain: Pulmonary embolism or aortic dissection?. [German]. *Intensivmedizin und Notfallmedizin* 2006;43(7):570-74.

77. Stanojlovic T, Pavlovic MP, Ciric-Zdravkovic SCZ, et al. P468 Utility of D-dimer testing in ruling out the diagnosis of acute aortic syndrome. *European Heart Journal Acute Cardiovascular Care* 2013;2(1)

78. Suzuki T, Katoh H, Watanabe M, et al. Novel biochemical diagnostic method for aortic dissection: results of a prospective study using an immunoassay of smooth muscle myosin heavy chain. *Circulation* 1996;93(6):1244-49.

79. Suzuki T, Distante A, Zizza A, et al. Preliminary experience with the smooth muscle troponin-like protein, calponin, as a novel biomarker for diagnosing acute aortic dissection. *European heart journal* 2008;29(11):1439-45. doi: <https://dx.doi.org/10.1093/eurheartj/ehn162>

80. Suzuki T, Distante A, Zizza A, et al. Diagnosis of acute aortic dissection by D-dimer: the International Registry of Acute Aortic Dissection Substudy on Biomarkers (IRAD-Bio) experience. *Circulation* 2009;119(20):2702-7. doi: <https://dx.doi.org/10.1161/CIRCULATIONAHA.108.833004>

81. Suzuki T, Trimarchi S, Sawaki D, et al. Circulating transforming growth factor-beta levels in acute aortic dissection. *Journal of the American College of Cardiology* 2011;58(7):775-75.

82. Tokuda N, Koga M, Ohara T, et al. Urgent detection of acute type a aortic dissection in ischemic stroke or TIA. *Stroke Conference: American Heart Association/American Stroke Association* 2016;47(SUPPL. 1)

83. Tokuda N, Koga M, Ohara T, et al. Urgent Detection of Acute Type A Aortic Dissection in Hyperacute Ischemic Stroke or Transient Ischemic Attack. *Journal of stroke and cerebrovascular diseases : the official journal of National Stroke Association* 2018;27(8):2112-17. doi: <https://dx.doi.org/10.1016/j.jstrokecerebrovasdis.2018.03.010>

84. von Kodolitsch Y, Schwartz AG, Nienaber CA. Clinical prediction of acute aortic dissection. *Archives of internal medicine* 2000;160(19):2977-82.

85. Wagner A, Domanovits H, Holzer M, et al. Plasma endothelin in patients with acute aortic disease. *Resuscitation* 2002;53(1):71-76.

86. Wang D, Wang Z-Y, Wang J-F, et al. Values of aortic dissection detection risk score combined with ascending aorta diameter >40 mm for the early identification of type A acute aortic dissection. *Journal of thoracic disease* 2018;10(3):1815-24. doi: <https://dx.doi.org/10.21037/jtd.2018.02.42>

87. Wang Y, Tan X, Gao H, et al. Magnitude of Soluble ST2 as a Novel Biomarker for Acute Aortic Dissection. *Circulation* 2018;137(3):259-69. doi: <https://dx.doi.org/10.1161/CIRCULATIONAHA.117.030469>

88. Weber T, Hogler S, Auer J, et al. D-dimer in acute aortic dissection. *Chest* 2003;123(5):1375-8.

89. Wei L, Faquan L, Jiumei C. Diagnostic mode for early diagnosis of acute aortic dissection. *Heart* 2012;2):E132.

90. Wiegand J, Koller M, Bingisser R. Does a negative D-dimer test rule out aortic dissection? *Swiss Medical Weekly* 2007;137(31-32):462.

91. Wilson S, Kinni H, Smoot T, et al. Overutilization of computed tomography angiography for acute aortic dissection: Identifying additional need for a reliable screening biomarker. *Academic Emergency Medicine* 2016;1):S56-S57.

92. Xiao Z, Xue Y, Yao C, et al. Acute Aortic Dissection Biomarkers Identified Using Isobaric Tags for Relative and Absolute Quantitation. *BioMed research international* 2016;2016:6421451. doi: <https://dx.doi.org/10.1155/2016/6421451>

93. Xu Z, Wang Q, Pan J, et al. Characterization of serum miRNAs as molecular biomarkers for acute Stanford type A aortic dissection diagnosis. *Scientific reports* 2017;7(1):13659. doi: <https://dx.doi.org/10.1038/s41598-017-13696-3>

94. Xu Z, Wei M, Guo X, et al. Changes of Serum D-Dimer, NT-proBNP, and Troponin I Levels in Patients with Acute Aortic Dissection and the Clinical Significance. *Evidence-Based Complementary & Alternative Medicine: eCAM* 2022;2022:8309505.

95. Xue C, Li Y. Value of D-Dimers in patients with acute aortic dissection. *Journal of Nanjing Medical University* 2007;21(2):86-88.

96. Yoshimuta T, Yokoyama H, Okajima T, et al. Impact of Elevated D-Dimer on Diagnosis of Acute Aortic Dissection With Isolated Neurological Symptoms in Ischemic Stroke. *Circulation journal : official journal of the Japanese Circulation Society* 2015;79(8):1841-5. doi: <https://dx.doi.org/10.1253/circj.CJ-15-0050>

97. Yuan S-M, Shi Y-H, Wang J-J, et al. Elevated plasma D-dimer and hypersensitive C-reactive protein levels may indicate aortic disorders. *Revista brasileira de cirurgia cardiovascular : orgao oficial da Sociedade Brasileira de Cirurgia Cardiovascular* 2011;26(4):573-81.

98. Zeng Q, Rong Y, Li D, et al. Identification of Serum Biomarker in Acute Aortic Dissection by Global and Targeted Metabolomics. *Annals of vascular surgery* 2020;68:497-504. doi: <https://dx.doi.org/10.1016/j.avsg.2020.06.026>

99. Zhang H, Yuan N, Guo J, et al. Comparisons of potential values of D-dimer and the neutrophil-to-lymphocyte ratio in patients with suspected acute aortic syndrome. *The American Journal of Emergency Medicine* 2023;69:44-51.

100. Zhang D, Zhao X, Wang B, et al. Circulating exosomal miRNAs as novel biomarkers for acute aortic dissection: A diagnostic accuracy study. *Medicine* 2023;102(30):e34474. doi: <https://dx.doi.org/10.1097/MD.0000000000034474>

101. Zhang H, Yuan N, Guo J, et al. Comparisons of potential values of D-dimer and the neutrophil- to-lymphocyte ratio in patients with suspected acute aortic syndrome. *American Journal of Emergency Medicine* 2023;69:44-51. doi: <https://dx.doi.org/10.1016/j.ajem.2023.03.059>

102. Zhao G, Zhao Y, Zhang H. Value of duration of chest pain, troponin, and D-dimer in differentiating acute high-risk chest pain patient. *Acta Medica Mediterranea* 2020;36(3):1587-91.

103. Zitek T, Hashemi M, Zagroba S, et al. A Retrospective Analysis of Serum D-Dimer Levels for the Exclusion of Acute Aortic Dissection. *Open Access Emergency Medicine* 2022;14:367-73.

104. Zheng Z, Zi Y, Jialin Y, et al. Value of D-dimer for detection of acute aortic dissection. *Heart* 2012;2):E268.
