# Supplementary material for: Diagnostic accuracy of the aortic dissection detection risk score alone or with D-dimer for acute aortic syndromes: Systematic review and meta-analysis
Source: PLoS One. 2024 Jun 21;19(6):e0304401. doi: 10.1371/journal.pone.0304401 (PMC11192411; doi:10.1371/journal.pone.0304401)
Supplement: S6 Appendix — (DOCX) [file pone.0304401.s006.docx]

**APPENDIX S6. QUADAS-2 quality assessment summary - Review authors’ judgements in detail**

|  | | | Chun & Siu, 2023[18] | Deng et al., 2023[19] | Gorla, 2017a[20] | Kodera, 2016[21] | Kotani, 2017[22] | McLatchie, 2023[23] | Morello, 2021[24] | Nazerian 2014a[25] | Nazerian 2014b[26] | Nazerian, 2018[27] | Ohle, 2019[28] | Rotella, 2018[29] | Yamashita, 2018[30] |
| --- | --- | --- | --- | --- | --- | --- | --- | --- | --- | --- | --- | --- | --- | --- | --- |
| **Patient selection domain** | | |  |  |  |  |  |  |  |  |  |  |  |  |  |
| 1. Was a consecutive or random sample of patients enrolled? | | | No | Yes | No | Unclear | Yes | No | Yes | Yes | Yes | Yes | Unclear | Unclear | Yes |
| 2. Was a case-control design avoided? | | | Yes | Yes | Yes | Yes | Yes | Yes | Yes | Yes | Yes | Yes | Yes | Yes | Yes |
| 3. Did the study avoid inappropriate exclusions? | | | Unclear | Yes | Unclear | Unclear | No | Yes | Yes | Yes | Yes | Yes | Unclear | Unclear | Yes |
| **SUMMARY: Risk of bias**  Could the selection of patients have introduced bias? | | | **High** | **Low** | **High** | **Unclear** | **High** | **High** | **Low** | **Low** | **Low** | **Low** | Unclear | Unclear | **Low** |
| **SUMMARY: Concerns regarding applicability**  Are there concerns that the included patients do not match the review question? | | | **Low** | **Low** | **Low** | **Low** | **Low** | **Low** | **Low** | **Low** | **Low** | **Low** | **Low** | **Low** | **Low** |
| **Index test domain: ADD-RS** | | |  |  |  |  |  |  |  |  |  |  |  |  |  |
| 4a. Were the index test results interpreted without knowledge of the results of the reference standard? | | | Unclear | Unclear | Yes | Yes | Yes | Unclear | Yes | Yes | Yes | Yes | Yes | Yes | Unclear |
| 5a. If a threshold was used, was it pre-specified? | | | Yes | Yes | Yes | Yes | Yes | Yes | Yes | Yes | Yes | Yes | Yes | Yes | Yes |
| **SUMMARY: Risk of bias**  Could the conduct or interpretation of the index test have introduced bias? | | | **Unclear** | **Unclear** | **Low** | **Low** | **Low** | **Unclear** | **Low** | **Low** | **Low** | **Low** | **Low** | **Low** | **Unclear** |
| **SUMMARY: Concerns regarding applicability**  Are there concerns that the index test, its conduct, or interpretation differ from the review question? | | | **Low** | **Low** | **Low** | **Low** | **Low** | **Low** | **Low** | **Low** | **Low** | **Low** | **Low** | **Low** | **Low** |
| **Index test domain: ADD-RS with D-dimer** | | |  |  |  |  |  |  |  |  |  |  |  |  |  |
| 4b. Were the index test results interpreted without knowledge of the results of the reference standard? | | |  |  | Yes | Yes | Yes | Unclear | Yes |  | Yes | Yes |  |  |  |
| 5b. If a threshold was used, was it pre-specified? | | |  |  | Yes | Yes | Yes | Unclear | Yes |  | Yes | Yes |  |  |  |
| **SUMMARY: Risk of bias**  Could the conduct or interpretation of the index test have introduced bias? | | |  |  | **Low** | **Low** | **Low** | **Unclear** | **Low** |  | **Low** | **Low** |  |  |  |
| **SUMMARY: Concerns regarding applicability**  Are there concerns that the index test, its conduct, or interpretation differ from the review question? | | |  |  | **Low** | **Low** | **Low** | **Low** | **Low** |  | **Low** | **Low** |  |  |  |
| **Reference standard domain** | | |  |  |  |  |  |  |  |  |  |  |  |  |  |
| 6. Is the reference standard likely to correctly classify the target condition? | | | Yes | Yes | Yes | Unclear | Yes | Yes | Yes | Yes | Yes | Yes | Yes | Yes | Yes |
| 7. Were the reference standard results interpreted without knowledge of the results of the index test? | | | Unclear | Unclear | Unclear | Unclear | Yes | Yes | Yes | Yes | Yes | Yes | Yes | Yes | Unclear |
| **SUMMARY: Risk of bias**  Could the reference standard, its conduct, or its interpretation have introduced bias? | | | **Unclear** | **Unclear** | **Unclear** | **Unclear** | **Low** | **Low** | **Low** | **Low** | **Low** | **Low** | **Low** | **Low** | **Unclear** |
| **SUMMARY: Concerns regarding applicability**  Are there concerns that the target condition as defined by the reference standard does not match the review question? | | | **Low** | **Low** | **Unclear** | **Unclear** | **Low** | **Low** | **Low** | **Low** | **Low** | **Low** | **Low** | **Low** | **Low** |
| **Flow and timing domain** | | |  |  |  |  |  |  |  |  |  |  |  |  |  |
| 8. Was there an appropriate interval between index test(s) and reference standard? | | | Unclear | Unclear | Unclear | Unclear | Unclear | Unclear | Yes | Yes | Yes | Yes | Unclear | Yes | Unclear |
| 9. Did all patients receive a reference standard? | | | Unclear | Yes | Unclear | Unclear | Unclear | No | Yes | Yes | Yes | Yes | Yes | Yes | No |
| 10. Did all patients receive the same reference standard? | | | Unclear | Unclear | Unclear | Unclear | Unclear | No | Unclear | Yes | Yes | Unclear | Yes | Yes | No |
| 11. Were all patients included in the analysis? | | | Yes | Yes | Yes | Yes | Yes | Unclear | Unclear | Yes | Yes | Yes | Yes | Yes | Yes |
| **SUMMARY: Risk of bias**  Could the patient flow have introduced bias? | | | **Unclear** | **Unclear** | **Unclear** | **Unclear** | **Unclear** | **High** | **Unclear** | **Low** | **Low** | **Unclear** | **Unclear** | **Low** | **High** |
|  |  |  | | | | | | | | | | | | | |
