# Supplementary material for: Diagnostic accuracy of the aortic dissection detection risk score alone or with D-dimer for acute aortic syndromes: Systematic review and meta-analysis
Source: PLoS One. 2024 Jun 21;19(6):e0304401. doi: 10.1371/journal.pone.0304401 (PMC11192411; doi:10.1371/journal.pone.0304401)

**S7 Appendix. Summary plots and forest plots for all meta-analyses**

**Figure S7-1: ADD-RS main analysis (N=12)**

**(a) Forest plot for ADD-RS>0**
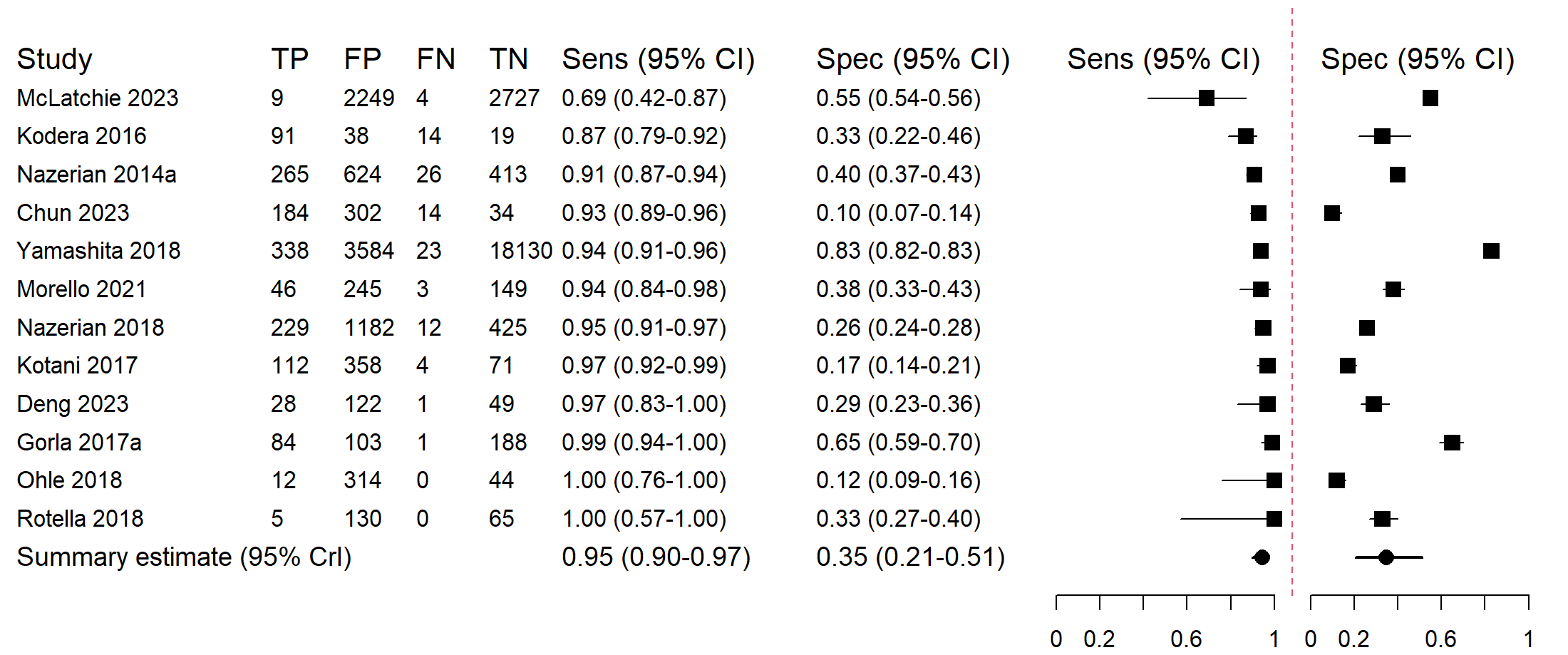


**(b) Forest plot for ADD-RS>1**
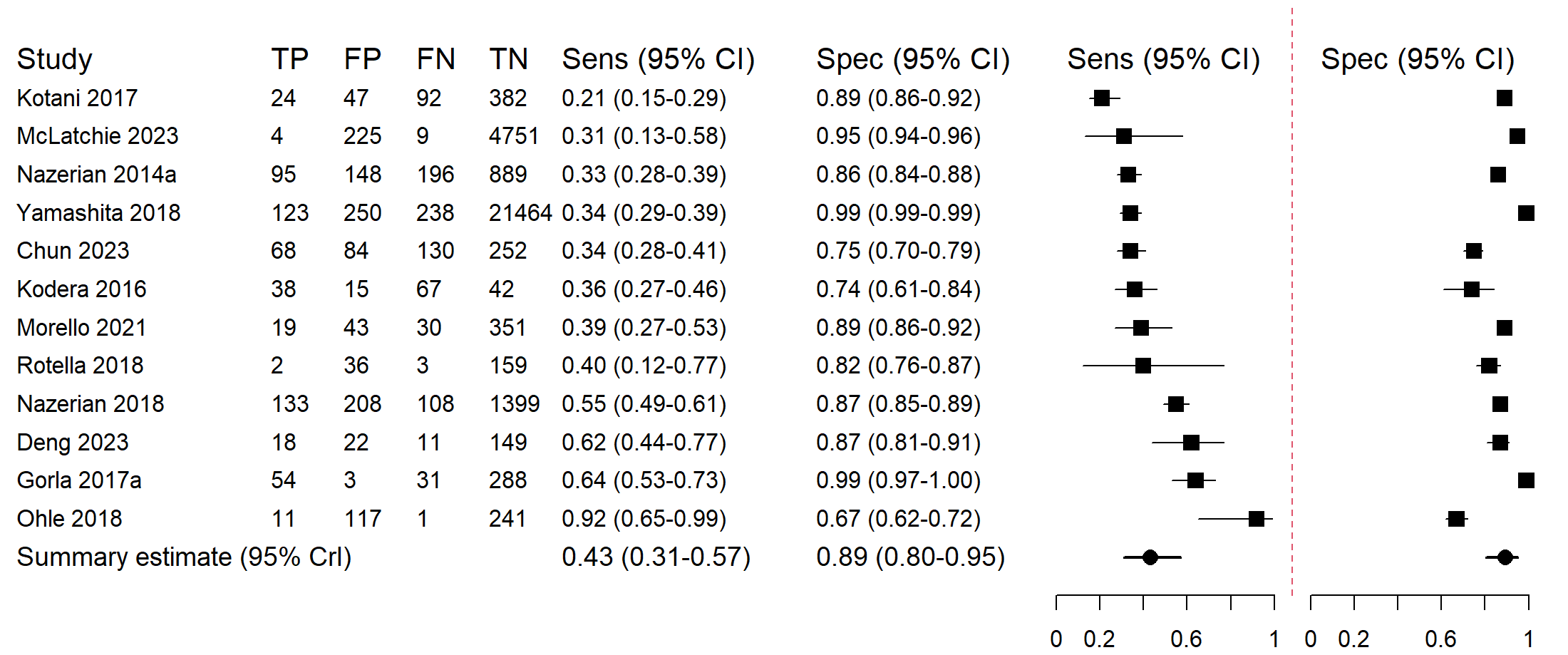


**Figure S7-2: ADD-RS with D-Dimer (N=6)**

**(a) Forest plot for ADD-RS>0 or D-Dimer>500ng/L**


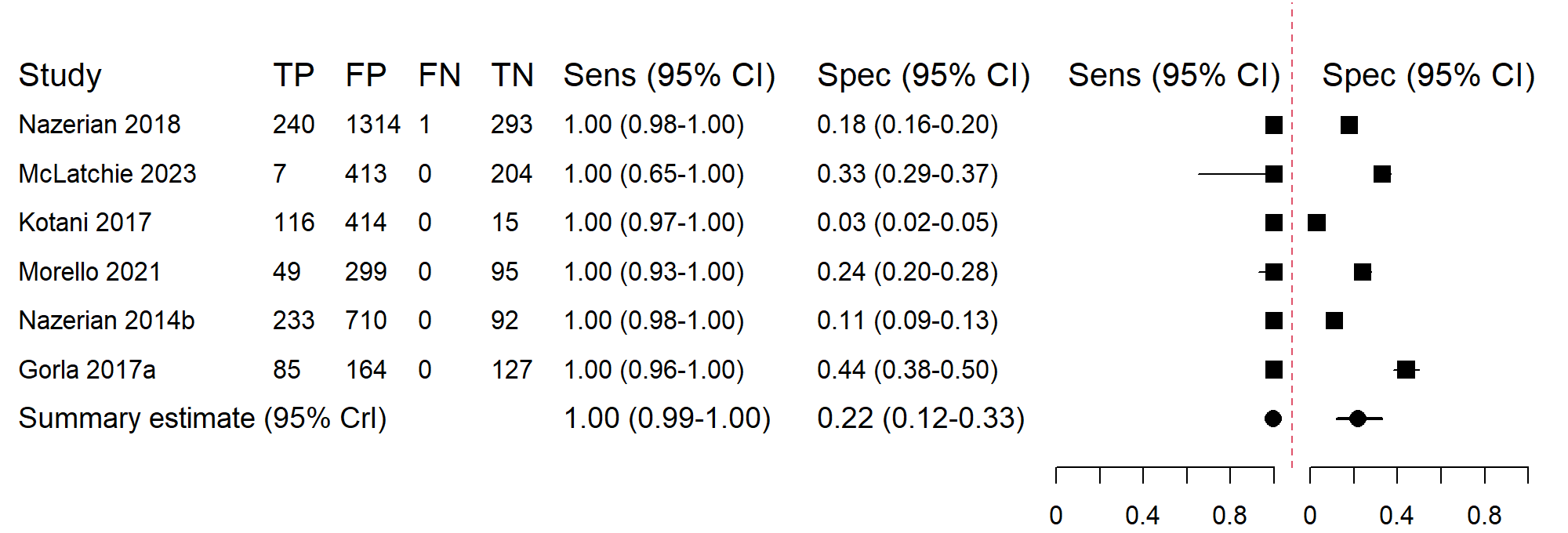


**(b) Forest plot for ADD-RS>1 or D-Dimer>500ng/L**


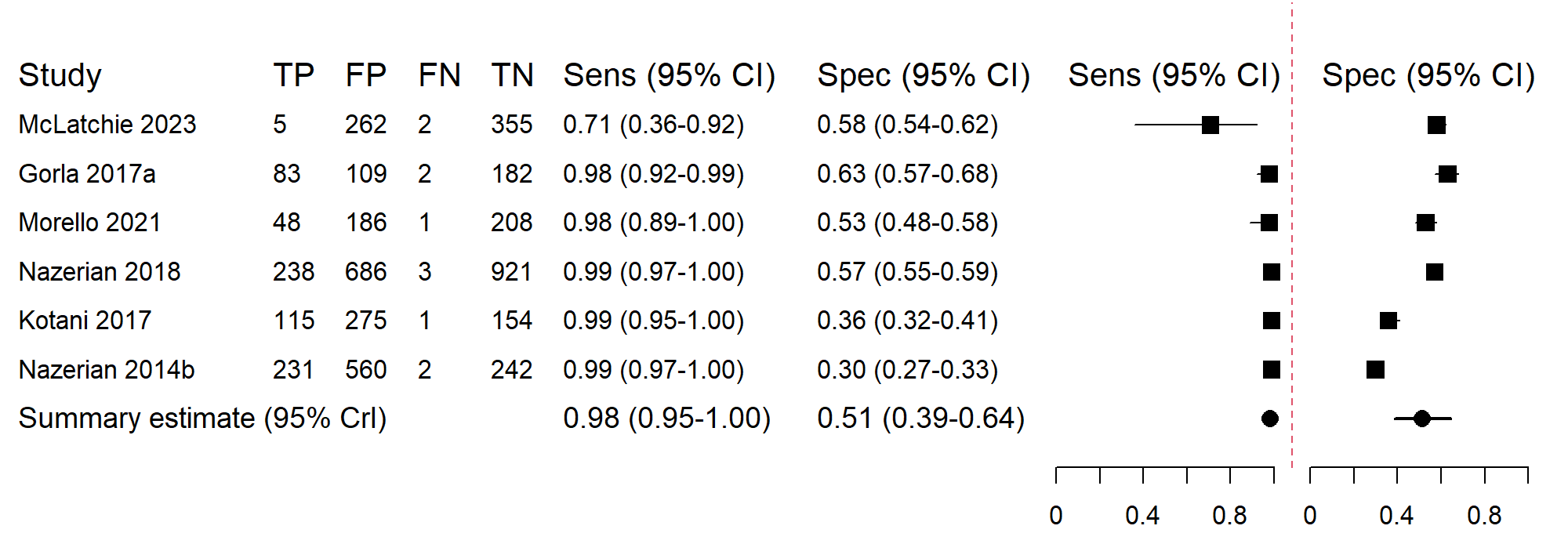


**Figure S7-3: Forest plot for Canadian guideline (N=6)**


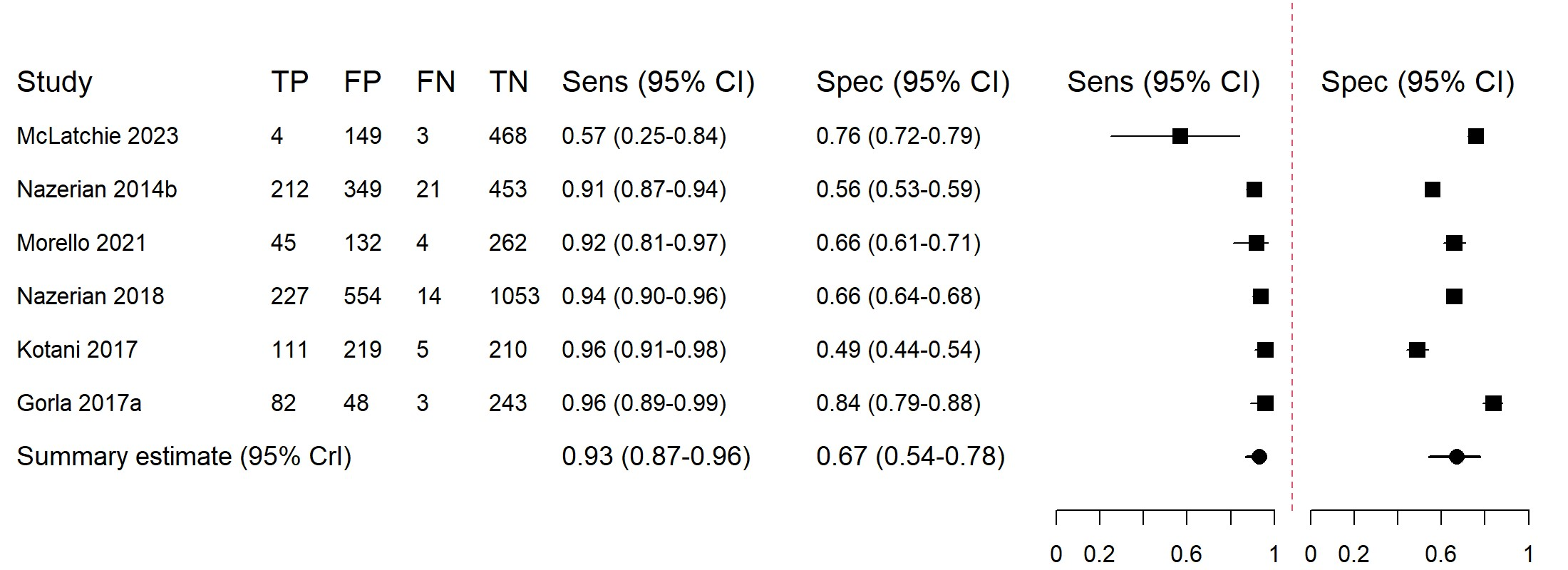


**Figure S7-4: ADD-RS sensitivity analysis (N=6)**

**(a) Summary plot**


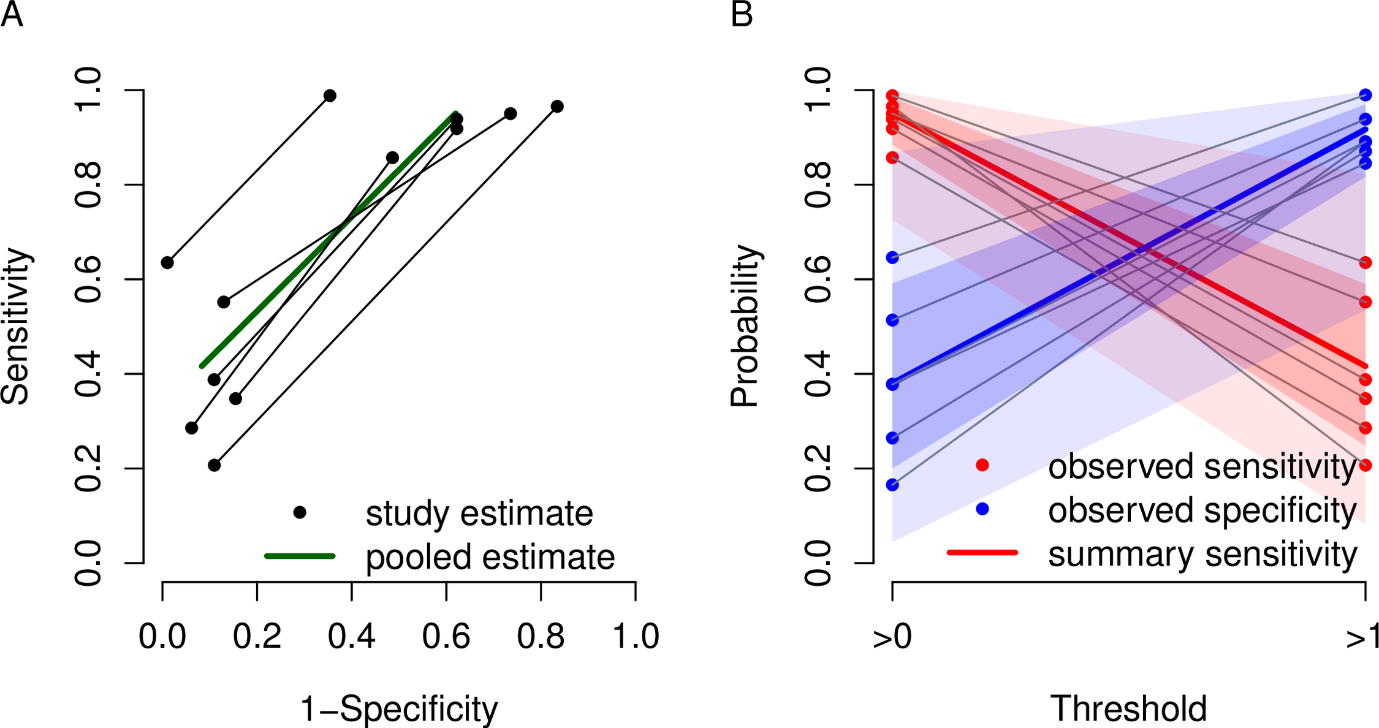


**(b) Forest plot for ADD-RS>0**


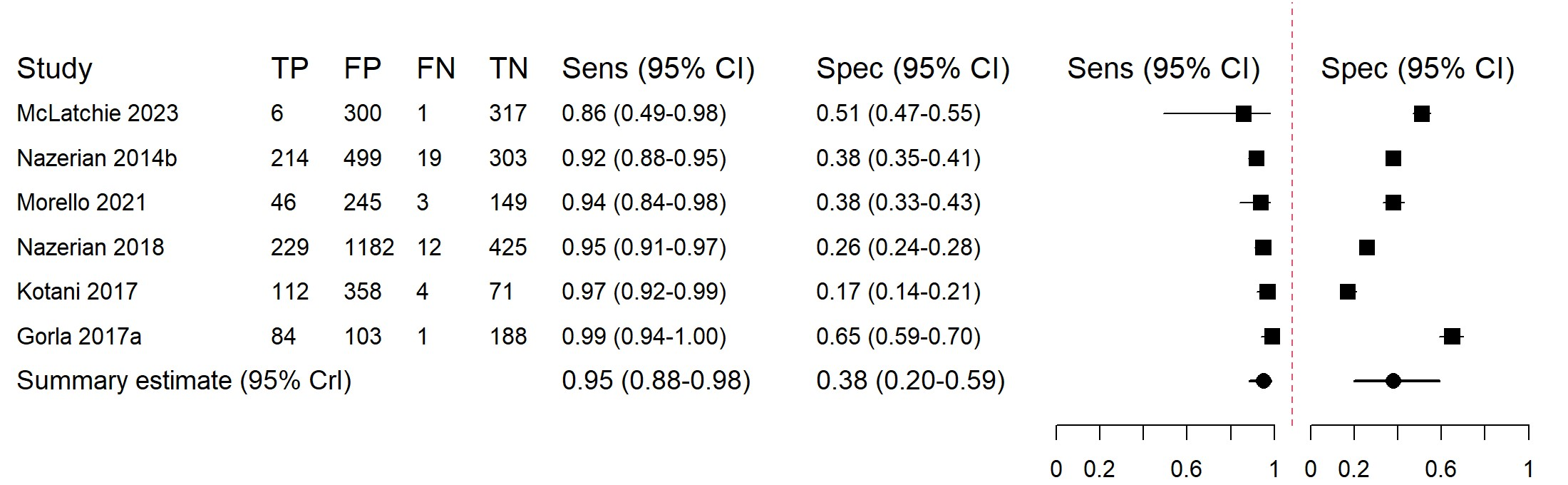


**(c) Forest plot for ADD-RS>1**


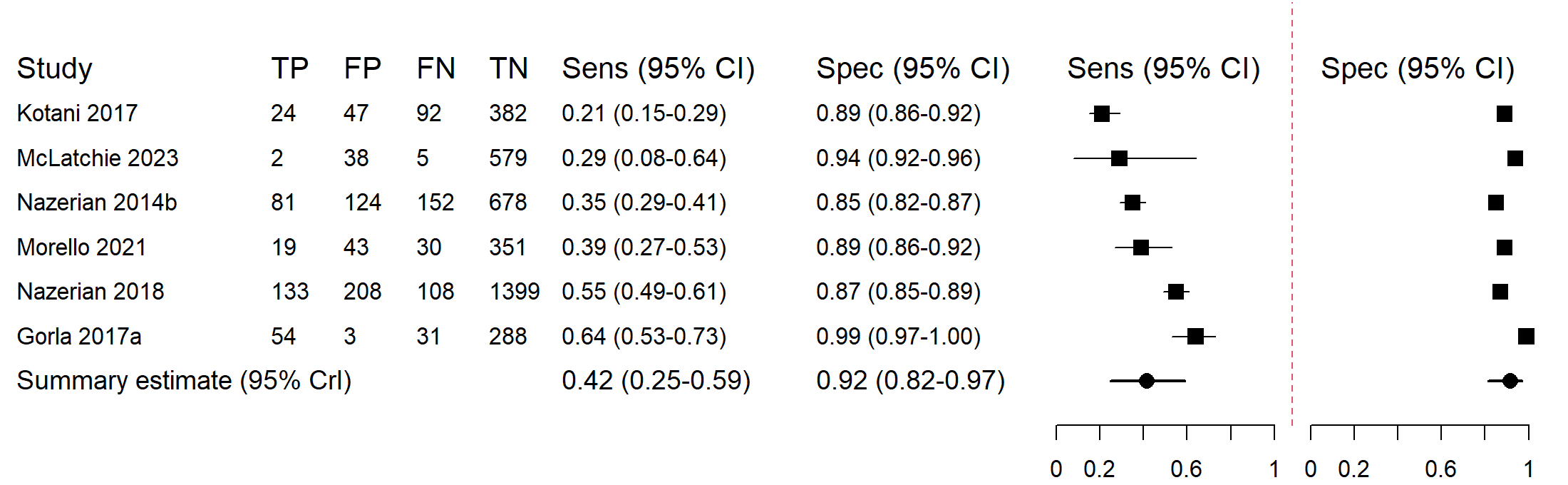

Supplement: S7 Appendix — (DOCX) [file pone.0304401.s007.docx]
