# Supplementary material for: Diagnostic accuracy of the aortic dissection detection risk score alone or with D-dimer for acute aortic syndromes: Systematic review and meta-analysis
Source: PLoS One. 2024 Jun 21;19(6):e0304401. doi: 10.1371/journal.pone.0304401 (PMC11192411; doi:10.1371/journal.pone.0304401)
Supplement: Attachment — Submitted filename: Response to Reviewers.docx [file pone.0304401.s009.docx]

**List of revisions and corrections addressing each of the concerns raised.**

| **Comments** | **Authors’ response** |
| --- | --- |
| **Journal requirements** | |
| 1. Please ensure that your manuscript meets PLOS ONE's style requirements, including those for file naming. | We have checked the style requirements and changed accordingly. |
| 2. Please remove any funding-related text from the manuscript and let us know how you would like to update your Funding Statement. | We have removed the funding statement and do not want to update it. |
| 3. Please include captions for your Supporting Information files at the end of your manuscript, and update any in-text citations to match accordingly. | We have included the captions for supporting information as requested. |
|  | |
| **Reviewer 1: The study is well-written. I have some major comments.** | |
| 1- Update your search from inception to January 2024. | We have updated the search and identified two additional studies that are now included in the analysis. |
| 2- Define abbreviations in their first use and only use abbreviated forms after definition (e.g., CTA in abstract). | We have made amendments as suggested. |
| 3- The structure of abstract is not appropriate. Use background/methods/results/conclusions headings. | We have made amendments as suggested. |
| 4- Remove subheadings from the discussion. | We have made amendments as suggested. |
| 5- The "strengths and limitations" subheading should be before conclusions. | We have made amendments as suggested. |
|  | |
| **Reviewer 2: The study is of utmost significance, although certain aspects require clarification.** | |
| 1- The p-value, AUC, and heterogeneity measures (e.g. I^2) are crucial outcomes of the Reitsma model. It is essential to include these values in the result for all analyses. | (1) We added the between study variance tau^2 as heterogeneity measure in the manuscript, and also added discussion about between study variance using prediction intervals.  (2) We did not calculate p-values for two reasons: (a) informative priors were used for pooling ADD-RS with D-Dimer>500 and Canadian guideline due to the limited number of studies identified, but not for ADD-RS alone; (b) Reitsma model was only used for Canadian guideline, not for the other two strategies, ADD-RS alone and ARR-RS with D-Dimer>500. The differences in the models and priors make the p-value less meaningful.  (3) We did not calculate AUC due to lack of data. For ADD-RS alone and ADD-RS with D-Dimer, there are only two different thresholds within all the included studies, which is not enough information to estimate a valid AUC. |
| 2- Certain sections of the manuscript require paraphrasing in order to prevent confusion and ensure the effective conveyance of information, thus necessitating proofreading. | Thanks for the suggestion. We have done proofreading. |
| 3- What was the author's rationale for excluding trauma patients and incidental patients from being eligible for inclusion in the study? | We excluded these patients because the ADD-RS was designed to be used in patients presenting with atraumatic chest pain. The primary studies do not include trauma patients and incidental patients. |
| 4- The systematic searches and subsequent findings must be updated, as it has been over a year since the last database search. | As above, we have updated the review and added two studies. |
| 5- Authors should ensure that search queries are provided in the supplementary data. | Yes, the search strategies are included in the appendix. |
| 6- The Scottish Intercollegiate Guidelines Network should be cited accurately. | We have added the citation as suggested. |
| 7- Authors should provide detailed explanations on how they justify the combining of various studies with different reference standard tests. | The reference standard tests used to diagnose AAS all have high accuracy for AAS. The main limitation is that some studies did not involve all patients receiving a definitive reference standard of imaging or operation/autopsy, and two studies (MacLatchie, Yamashita) had a large proportion receiving no definitive reference standard. We chose not to exclude these studies because studies in which all patients have definitive imaging are likely to involve a more selected population with higher prevalence, so findings may not be generalisable to a lower risk population. We note in the limitations section that our inclusive selection criteria may contribute to the observed heterogeneity. |
| 8- In table 3, the authors should provide an explanation for the presence of two columns dedicated to Sensitivity and Specificity in the table's footnote. | We have amended the table 3 to have one column for pooled sensitivity and one column for pooled specificity. |
| 9- The discrepancy in the figure representation of the "Summary plot for Canadian guideline based strategy analysis (N=6)" hinders its comparability. Authors should provide an explanation for this decision or modify of the figure. | Thanks for your suggestion. We have included an additional figure (Fig 5) to present the pooled estimates and their 95% credible intervals. The discrepancy in the figure is caused by the nature of data. For ADD-RS alone and with D-Dimer>500, more than one threshold is analysed jointly with a multinomial meta-analysis model that takes account of the dependency of test results on the value of threshold. The results are presented along with the test threshold. For the Canadian guideline with only one threshold involved, a bivariate model is used instead, and the results are presented in one plot. |
| 10- It is recommended to perform a meta-regression analysis considering age and the percentage of females due to the nature of the subject and the potential effects of these covariates. | We are unable to perform meta-regression to explore the sources of heterogeneity among the included studies due to not all included studies reported study level age and percentage of female. |
